# Improved insect‐proofing: expressing double‐stranded RNA in chloroplasts

**DOI:** 10.1002/ps.4870

**Published:** 2018-03-07

**Authors:** Julia Bally, Elane Fishilevich, Andrew J Bowling, Heather E Pence, Kenneth E Narva, Peter M Waterhouse

**Affiliations:** ^1^ Centre for Tropical Crops and Biocommodities QUT, Brisbane, QLD Australia; ^2^ Dow AgroSciences Indianapolis IN USA

**Keywords:** Trans, Kingdom, Chloroplast, RNAi

## Abstract

RNA interference (RNAi) was discovered almost 20 years ago and has been exploited worldwide to silence genes in plants and animals. A decade later, it was found that transforming plants with an RNAi construct targeting an insect gene could protect the plant against feeding by that insect. Production of double‐stranded RNA (dsRNA) in a plant to affect the viability of a herbivorous animal is termed trans‐kingdom RNAi (TK‐RNAi). Since this pioneering work, there have been many further examples of successful TK‐RNAi, but also reports of failed attempts and unrepeatable experiments. Recently, three laboratories have shown that producing dsRNA in a plant's chloroplast, rather than in its cellular cytoplasm, is a very effective way of delivering TK‐RNAi. Our review examines this potentially game‐changing approach and compares it with other transgenic insect‐proofing schemes. © 2018 The Authors. *Pest Management Science* published by John Wiley & Sons Ltd on behalf of Society of Chemical Industry.

## 1 INTRODUCTION

A significant constraint to crop production is the damage caused by insects, with arthropod pests causing global crop losses of 13–18%, with an estimated (in 2014) value loss of US$470 billion annually.^1^ This has provided strong motivation for the development of insect‐resistant crops, and transgenesis is a very effective way of producing them. Currently, all of the commercialized insect resistance traits for lepidopterans are based on insecticidal proteins derived from the insect pathogenic bacterium *Bacillus thuringiensis* (Bt).^2^ While this technology has been very effective, field selection for resistant insect populations threatens its long‐term durability.^3^ Therefore, alternative and complementary insect control technologies are being sought.

Over the past two decades, there has been intensive research into understanding RNA interference (RNAi), an endogenous gene regulation and defense mechanism, in plants and animals. The mechanism has been harnessed to confer insect protection in transgenic plants.^4^, ^5^ To achieve this, double stranded (ds) RNA was produced in the plant which guided the RNAi machinery in the herbivorous insect to silence one of its own essential genes. This production of dsRNA in a plant affecting the viability of an insect is termed trans‐kingdom RNAi (TK‐RNAi). There have been many additional examples of insects being stunted or killed by RNAi and TK‐RNAi, but not all insect species respond equally^6^, ^7^, nor do the results always appear repeatable. 8 However, producing the guide dsRNA in a plant's chloroplasts rather than in its cellular cytoplasm has recently been shown to enhance the effectiveness of TK‐RNAi.^9^, ^10^, ^11^, ^12^ Here, we place chloroplast‐based (cp) TK‐RNAi in context among other transgenic insect‐proofing approaches.

## 2 RNAI, ENVIRONMENTAL RNA AND TRANS‐KINGDOM RNAI

TK‐RNAi utilizes the production of long dsRNA, long self‐complementary hairpin (hp) RNA or small interfering (si) RNA in an organism of one kingdom to cause an exogenous effect by guiding the RNAi machinery in an organism of another kingdom. Both TK‐RNAi and the delivery of dsRNA in artificial diets to animals fall under the umbrella term: environmental RNAi (eRNAi). The most common TK‐RNAi approach has been making stable transgenic plants that express hpRNA‐containing sequences, as complementary “arms”, derived from essential insect genes. The expectation is that an insect feeding on such a plant will ingest hpRNA and the derived siRNAs, which will direct the insect's RNAi machinery to degrade mRNA of the target gene. This has the effect of impairing the insect's viability, development, and/or fecundity. The target genes, and sequences therein, have usually been selected using results from experiments in which the target insect is injected or fed (in artificial diet) with different dsRNA sequences. Unfortunately, the conformation and amount of dsRNA that reaches the cells lining the gut of an insect feeding on a transgenic plant expressing hpRNA are less measurable and predictable than with artificial diet or injection assays. A further consideration is whether the evoked RNAi is restricted to the insect's gut cells taking up the dsRNA (cell autonomous RNAi) or also spreads to other cells (systemic RNAi).^6^, ^13^, ^14^ Both plant and insect cells have endogenous RNAi pathways but they differ in important ways that, as discussed below, affect the processing and the outcome of TK‐RNAi. Often, hpRNA and dsRNA are treated as synonymous. However, if two separate but complementary RNA strands are produced from separate transcription units within the same cell, the transcripts must find each other and anneal to form dsRNA. This is thermodynamically much less likely than a single‐stranded RNA, with self‐complementarity (i.e. an hpRNA), folding back and hybridizing with itself to form a duplex structure. Furthermore, dicers may process the duplex structure at the open end of an hpRNA at a different rate from the region at the closed “end”, and hpRNA transgenes appear more vulnerable to gene silencing than two non‐adjacent sense and antisense transgenes. Therefore, in this review, we maintain a distinction between these two forms of duplexed RNA.

### 2.1 RNAi machinery: plant cell versus insect cell versus chloroplast

**Figure 1:**
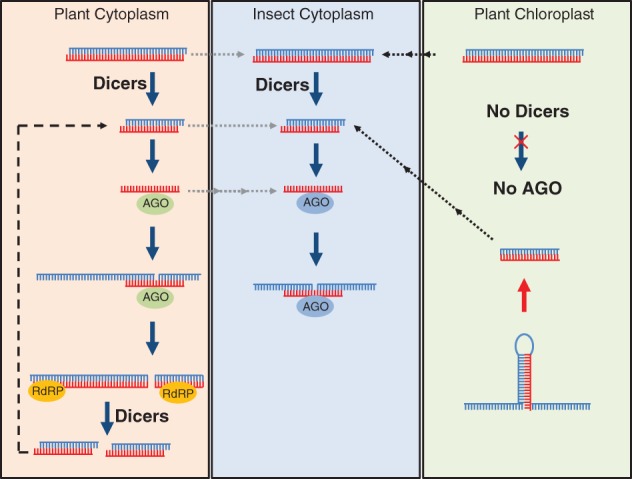
Parallel RNA silencing pathways in the plant cytoplasm, insect cytoplasm and plant chloroplast. In the plant cytoplasm, delivered dsRNA or hpRNA is processed by Dicer enzymes into primary siRNAs, which in turn direct AGO1 to their target mRNAs. Silencing amplification producing secondary siRNAs is then driven by RdRps. This amplification mechanism is absent in insect cells because of the lack of RdRps. In plant chloroplasts, as there is no Ago and DCL activity, dsRNA delivered into this compartment are not processed.

The endogenous gene silencing capability of a plant cell has many different facets that may be regarded as a suite of overlapping pathways, including the RNAi pathway (duplexed RNA‐induced targeted RNA degradation). These pathways control the expression of developmentally regulated genes, repress the activity of repetitive elements in the plant genome, and provide resistance against invading nucleic acids such as viruses.^15^ They are mediated by four (in dicots) or five (in monocots) different Dicer‐like RNase III‐like endonucleases (DCLs) that collectively process duplexed RNA into 21‐, 22‐, and 24‐nt siRNAs, and appropriate highly structured RNA transcripts into ∼21‐nt microRNAs.^15^, ^16^, ^17^ The siRNAs and microRNAs are loaded onto specific effector proteins of the multi‐member Argonaute (AGO) family that have RNA cleavage or binding capability.^18^, ^19^ In the RNAi pathway, DCL2 and DCL4 produce siRNAs that predominantly guide AGO1 and AGO7^20^ which cleave target RNAs. Further, siRNAs are generated through a process that involves siRNAs, target RNAs, and at least one RNA‐directed RNA polymerase, RDR6 (Fig. 1).

In an insect cell, RNAi starts by cleavage of dsRNA by Dicer‐2 into ∼21‐nt siRNAs^21^, which are loaded onto AGO2.^22^ As with plant siRNAs and their associated AGO1^23^, one of the siRNA strands (the passenger strand) is then degraded while the other (the guide strand) remains associated with AGO2 and mediates recognition and cleavage of RNA sequences complementary to the guide strand (Fig. 1).^13^, ^24^ Unlike plants, fungi, and nematodes, insects do not possess a canonical RNA‐dependent RNA polymerase (RdRp), suggesting that amplification of RNAi may not occur in these animals.^25^

Chloroplasts do not have RNAi machinery. Their genome encodes about 130 genes but none is Dicer‐like or Argonaute‐like, nor is there any evidence suggesting import of nuclear‐encoded DCL or AGO proteins. With this lack of core RNAi enzymes, it is widely believed that chloroplasts do not have an RNAi pathway (Fig. 1).

### 2.2 RNAi responses in insects

The majority of agronomically important insects, particularly for row crops, fall within the orders of Lepidoptera, Coleoptera, and Hemiptera. They each possess the RNAi hallmark genes Dcr‐2 and AGO2, and both lepidopteran and coleopteran cells appear able to take up dsRNA^26^, but these three orders have stark differences in their capacity for gene suppression by eRNAi. The response of Coleoptera is highly efficient.^6^, ^27^, ^28^, ^29^ For example, the Colorado potato beetle, Leptinotarsa decemlineata 4, 28, and the western corn rootworm, Diabrotica virgifera virgifera, show robust eRNAi^4^, ^30^ in response to extremely low levels (< 10 ppb) of dsRNA in an artificial diet.^31^ However, Lepidoptera generally show weaker and more variable eRNAi responses and require higher levels (1 x 10^2^ to 1 x 10^4^ ppm (31)) of dsRNA to initiate them.^8^ Some hemipteran pests, especially the plant bugs and stink bugs, are even more recalcitrant to eRNAi.^27^, ^32^, ^33^ In studies using different target genes, eRNAi was effective against 49% of 290 genes from Coleoptera^34^ and 38% of 130 genes from Lepidoptera.^8^ eRNAi efficacy increases with dsRNA length in the Dipteran Drosophila melanogaster 35, and >70 bp dsRNA is necessary for oral RNAi in Coleoptera,^36^, ^37^, but there is no such trend in Lepidoptera.^8^

It should be noted that these examples and trends are taken from a spectrum of results, including some where seemingly similar experiments by different laboratories have produced very different conclusions.^8^ Nevertheless, the choice of target gene, length of dsRNA, method of dsRNA delivery, and capacity of the target species to traffic dsRNA all appear to be factors in the strength of eRNAi outcome.

### 2.3 Insect nucleases and dsRNA trafficking

The ways in which insects protect themselves against viruses may be linked to differences in their capacity for eRNAi and sensitivity to dsRNA induction. Coleopterans have an active dsRNA uptake and processing mechanism, probably reflecting a defense strategy of cellular dsRNA uptake coupled to an efficient RNAi response, and hence a strong induction of the eRNAi mechanism against viruses such as alphanodaviruses. Indeed, the nodaviruses encode RNAi suppressor proteins.^38^ The spectrum of eRNAi levels in Lepidoptera is possibly linked to the range of extracellular nuclease activity in their digestive tracks and hemolymph^39^, ^40^, and hence their capacity to degrade dsRNA that would otherwise be absorbed into gut or hemoceol cells to trigger RNAi.^41^, ^42^ Hemiptera (especially plant bugs and stink bugs) defend against viruses and dsRNA extra‐orally by injecting their nuclease‐containing^43^, ^44^ saliva into plant tissues prior to feeding. Consistent with this hypothesis, managing hemipteran insect pests, especially stinkbugs like Halyomorpha halys (Stål) (Heteroptera: Pentatomidae), the brown marmorated stinkbug (BMSB), using RNAi technology has posed a tremendous challenge.^27^, ^32^, ^33^ However, eRNAi responses have been reported across a diverse range of hemipterans^37^, ^45^, including aphids (Hemiptera: Aphididae)^46^, ^47^, ^48^, ^49^, ^50^, ^51^, ^52^, ^53^, ^54^, psyllids^55^, leafhoppers^56^, planthoppers^57^, and whitefly.^58^, ^59^, ^60^ Perhaps the saliva of most plant‐feeding hemipterans, which inject salivary enzymes into the plant's tissues (mesophyll, xylem and/or phloem), has lower dsRNase activity than that of hemipterans that are seed feeders or predators.^44^

Recent work has identified a further compelling reason why it is easier to obtain efficient eRNAi in Coleoptera than in Lepidoptera. Tagged dsRNA was efficiently taken up by cell lines from these insects, but the dsRNA was only processed into siRNA, and target mRNA degraded, in the coleopteran cells. Similarly, siRNAs could be detected in coleopterans D. v. virgifera and L. decemlineata but not in lepidopterans Spodoptera frugiperda and H. zea when fed on plants expressing hpRNA.^61^ A current model is that dsRNA is taken up into lepidopteran cells and retained in endocytic compartments of the cells, whereas dsRNA taken up into coleopteran cells escapes these compartments and is exposed to, and processed by, the RNAi machinery.^26^

### 2.4 Amplification and spread of RNAi

In plant or Caenorhabditis elegans cells, dsRNA is not only processed into siRNAs but also amplified via the action of RdRps^20^, ^62^, ^63^, ^64^, in a process that uses the target mRNA as a template for the synthesis of more dsRNA, and thereby siRNAs (Fig. 1). This enhances and perpetuates the RNAi, after initiation, and facilitates its spread into surrounding cells. However, in honeybees and Drosophila, RNAi can be maintained for weeks without the involvement of a canonical RdRp, leading to the hypothesis that an amplification process can occur by a different mechanism.^65^ In Drosophila this involves a subunit of DNA‐dependent RNA polymerase II (Pol II).^66^

**Figure 2:**
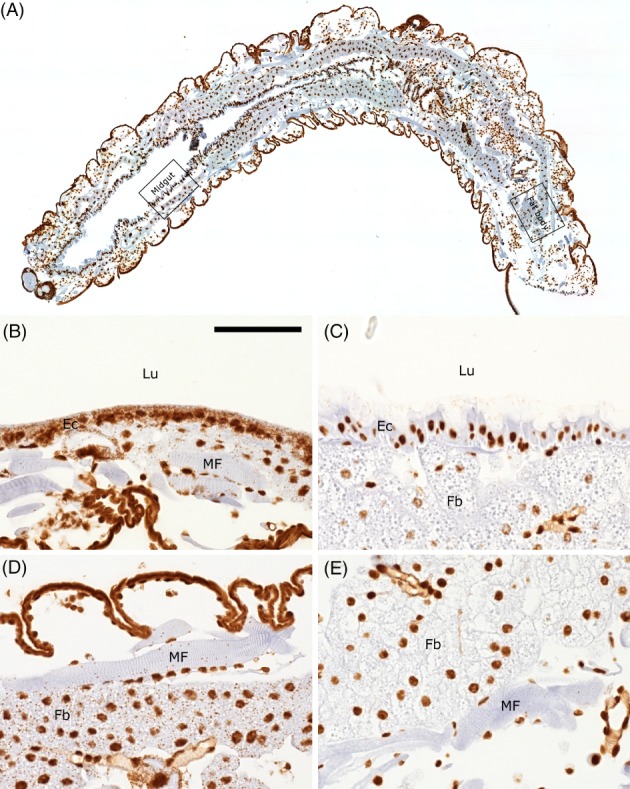
Characterization of RNAi spread in D. v. virgifera larva using single‐molecule RNAscope in situ hybridization to detect V‐ATPase C. (A) An overview image of an entire first‐instar D. v. virgifera larva longitudinal section probed with an irrelevant bacterial gene (RNAscope probe dihydrodipicolinate reductase (dapB) DapB; negative control). The anterior portion of the larva is to the left, and the posterior is to the right. Regions representative of the midgut regions shown in panels (B) and (D), and posterior fat body regions shown in (C) and (E) are indicated with boxes. (B) A representative region of the anterior midgut, probed for V‐ATPase C mRNA (small brown speckles). Before the larvae have fed on dsRNA, a large number of V‐ATPase C mRNAs are present in the midgut enterocytes, and a smaller number are present in fat body and muscle fiber cells. Background signal in nuclei and cuticle is represented by Brown diaminobenzidine(DAB) staining. (C) After 48 h of feeding on diet containing V‐ATPase C‐specific dsRNA, the amount of V‐ATPase C mRNA in the enterocytes of the anterior midgut is dramatically reduced. Only a few faint mRNA speckles remain. (D) Before feeding V‐ATPase C dsRNA, the fat body and, to a lesser extent, the muscle fiber cells in the posterior portion of the larva contain V‐ATPase C mRNA. (E) After feeding on V‐ATPase C dsRNA for 48 h, the level of V‐ATPase C mRNA in the posterior fat body and muscle fibers is reduced nearly to zero, clearly demonstrating systemic spread of RNAi in this animal. Lu, lumen; MF, muscle fibers; EC, enterocytes; Fb, fat body. Scale bar = 50 μm; panels (B), (C), (D), and (E) are at the same magnification. This illustration is a high‐magnification image of a sister section from the study by Li et al. 70. The section was prepared and probed as described in Li et al. 70

It is also possible that RNAi can spread without the need for amplification, if sufficient dsRNA is continuously taken up. Indeed, single high‐dose feeding of dsRNA was less effective than continuous diet‐based application in Coleoptera.^67^, ^68^, ^69^ There is clear evidence that the RNAi response can be systemic in insects lacking a canonical RdRp.^8^, ^37^, ^70^ For example, in situ hybridization studies of D. v. virgifera fed on dsRNA demonstrate that target mRNA is degraded in tissues distal from the midgut.^70^, ^71^ This is illustrated in Fig. 2, which is a high‐magnification image of a sister section from the study by Li et al. 70

### 2.5 Conformation of dsRNAs in TK‐RNAi plants

The protection of maize plants from the western corn rootworm, *D. v. virgifera* 4, and protection of *Arabidopsis thaliana* plants from the cotton bollworm, *Helicoverpa armigera* 5, are two early examples of plant–insect TK‐RNAi. The nuclear genomes of these plants were transformed with hpRNA constructs targeting an insect *vacuolar ATPase* (*V‐ATPase*) and a *p450 monooxygenase* gene, respectively. Transcribed hpRNAs were rapidly processed by endogenous RNAi machinery in the plant cell, producing 21‐, 22‐, and 24‐nt siRNAs^72^, as described in Section 2.1 and in Fig. 1. To explain how these two research papers (4, 5) were reporting successful TK‐RNAi after years of anecdotes of failure by others using similar approaches, it was suggested that their plants were transcribing hpRNA faster than it was being processed into siRNAs.^25^ This would provide the feeding insects with a quantity of long hpRNA, whereas transgenic plants with less active hpRNA transcription may only be presenting siRNAs to herbivorous insects. In injection and artificial diet studies, siRNA is usually delivered in a duplexed form, as used in mammalian experiments. However, in hpRNA‐expressing plants, the predominant form of accumulating siRNA is single‐stranded and associated with an AGO protein^15^; this seems unlikely to be efficiently dissociated from the plant AGO and installed *de novo* onto an insect AGO.

## 3 CHLOROPLAST‐DELIVERED TK‐RNAI

**Figure 3:**
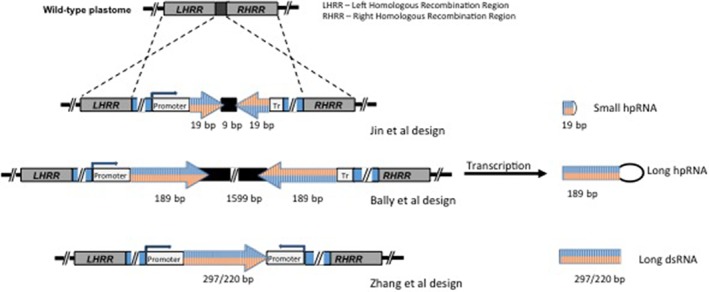
Schematic representation of transformation vectors for dsRNA expression from the plastid genome used in the three reports of cpTK‐RNAi. The targeted plastid genome region is represented. The left homologous recombination region (LHRR) and right homologous recombination region (RHRR) are the left and right plastid recombination regions, respectively, present in all transforming vectors. The cassettes have been designed to produce three different types of dsRNA: small hpRNA, long hpRNA and long dsRNA.

The chloroplast plastome consists of a double‐stranded circular DNA molecule with a prokaryotic type of organization. It is highly polyploid, and typically contains approximately 130 genes, including those coding for chloroplast ribosomal and transfer RNA, and some of the proteins involved in photosynthesis. Most proteins in the chloroplast are imported from the cytoplasm and are encoded by the nuclear genome. Engineering the chloroplast genome was pioneered in the 1980s and was enabled by the invention of the gene gun.^73^ Bombarding leaf pieces with micro particles, coated with transforming DNA, facilitates transplastomic integration. The transforming DNA encodes an antibiotic resistance gene and the gene of interest, both regulated by chloroplast promoters. This two‐gene cassette is flanked by sequences homologous to a region of the plastome to facilitate specific insertion into the plastome by homologous recombination (Fig. 3). The bombarded leaf tissue is placed on medium containing the appropriate antibiotic and regeneration‐promoting hormones. As the tissue regenerates into new plants, the antibiotic selection is maintained until all the wild‐type chloroplasts are replaced with transformed ones. This makes the regenerated plants, including their chloroplasts, genetically identical. In contrast, conventional nuclear transformation using *Agrobacterium tumefaciens* inserts the new DNA at random locations in the plant genome and produces a library of transformants with differing levels of expression dependent on the insertion sites of the transgenes.

### 3.1 The three reports of cpTK‐RNAi

Chloroplast transformation has extraordinary potential for high‐level expression of the transgene and accumulation of foreign proteins, as a consequence of the polyploidy of the plastid genome. For example, the insecticidal toxin Bt Cry2A accumulated to 46% of total soluble protein when this gene was localized in the plastome of tobacco.^74^ Amazingly, this had no obvious morphological effect on the plant.^74^ Therefore, when high levels of dsRNA are required for better insect protection, such levels may be readily attained in the chloroplast compartment without perturbation of the plant metabolic system.^75^ Three recent studies, each taking a slightly different approach (Fig. 3), have reported protection against insects by generating plants expressing hp/dsRNA in their chloroplasts.^10^, ^11^, ^12^ Two of them obtained protection of tobacco species against a lepidopteran^10^, ^12^ and one protected tobacco and potato against a coleopteran.^11^ In the coleopteran work, dsRNA was generated in chloroplasts by insertion of genetic constructs with convergent promoters flanking a 450‐bp fragment of a *β‐actin* (*ACT*) gene, a 380‐bp fragment of a transcript encoding a membrane‐remodeling protein, or a concatenation of the two fragments.^11^ The transplastomic plants produced large amounts of unprocessed dsRNA and greatly inhibited the growth and development of Colorado potato beetles, *L. decemlineata,* feeding on their leaves. The most potent insecticidal activity was displayed by the transplastomic potato plants expressing dsRNA against ACT, which caused 100% mortality of first‐instar larvae within 5 days of feeding. One of the lepidopteran studies expressed a 189‐nt hpRNA, containing an *acetylcholinesterase* sequence, in the chloroplasts of *Nicotiana benthamiana*. 10 These plants accumulated high levels of unprocessed hpRNA and significantly retarded the growth of *H. armigera* feeding on them.^10^ Both studies generated plants with counterpart constructs transformed into their nuclear genomes; these accumulated low levels of intact dsRNA or hpRNA, produced abundant siRNAs, and displayed little or no pesticidal activity. The other lepidopteran study generated *Nicotiana tabacum* plants expressing a short (∼19‐nt arm) hpRNA against the *cytochrome p450 monooxygenase, V-ATPase, or chitin synthase* gene in their chloroplasts. Although no comparison was made with nuclear transformed counterparts, the plants were strongly protected against herbivory by *H. zea*.^12^, ^76^ In all three studies, the sequences used to generate dsRNA or hpRNA were chosen to be specific to the target species and are very unlikely to have adverse effects on mammals.

### 3.2 Limitations in making cpTK‐RNAi plants

These recent studies demonstrate the efficacy of producing pesticidal dsRNA in chloroplasts. For lepidopteran and hemipteran pests, it should be an attractive way to ensure sufficiently high doses of intact dsRNA for effective TK‐RNAi. However, the approach is not without its challenges. Nuclear transformation is routine for most crop species but transformation of their chloroplasts is more difficult.

A number of transplastomic crops have been generated, including soybean (*Glycine max*)^77^, potato (*Solanum tuberosum*)^11^ *,* tomato (*Solanum lycopersicum*)^78^, and carrot (*Daucus carota*)^79^ but, with the exception of rice, which gave unstable and heteroplasmic plants^80^, plastid transformation of cereal crops has not been achieved. The main challenge lies in selecting for cells with transgenic chloroplasts during the induction of somatic embryogenesis. This is especially difficult if the starting material is not green because pro‐plastids in such tissues have different gene expression and regulation from those in mature chloroplasts.

## 4 FUTURE DIRECTIONS

The increased RNAi response seen with plastid‐based dsRNA delivery in both coleopteran^11^ and lepidopteran^10^, ^12^ insects stems from a combination of beneficial, possibly essential, factors. The expression in chloroplasts gives both a sustained high level of dsRNA (needed for non‐amplified eRNAi) and the preservation of intact long dsRNA. Additionally, encapsulation of dsRNA within the chloroplast membranes may provide physical protection against insect digestive systems. The concept of cpTK‐RNAi promises to be a game‐changer for insect protection. However, this excitement is based on only three examples. It will be interesting to see if the approach is rapidly adopted and expanded to other pest species and other crops. It seems likely that cpTK‐RNAi will be readily usable to protect solanaceous crops against coleopteran pests, given the 100% mortality of Colorado beetle feeding on cpTK‐RNAi potatoes. For this application, the questions to be addressed are: 1. Which genes are the most efficacious targets? 2. Is an hpRNA better or worse than dsRNA in terms of efficacy of cpTK‐RNAi and stability of the construct within the chloroplast genome? 3. Is the technology robust enough on its own for use in the field or is it better as part of a two‐pronged attack? It has already been demonstrated that nuclear‐expressed TK‐RNAi in combination with expression of Bt proteins is a very effective strategy for protecting maize against the coleopteran *D. v. virgifera* 81, 82 and has the capacity to control insects developing field resistance to Bt proteins.^83^ Multiple genes can be targeted simultaneously with cpTK‐RNAi by ds/hpRNAs comprised of concatenated sequences. This raises the possibility of deploying multi‐targeted cpTK‐RNAi in combination with Bt, which may be even more effective and durable in the field.

There are many more questions about cpTK‐RNAi of lepidopteran insects. eRNAi has been reported to be operative in many different lepidoptera species, but in most cases the efficacy has been low or highly variable depending on researchers, tissues, timing and delivery method.^8^ Yet two groups have shown a pesticidal effect against *Helicoverpa* species using cpTK‐RNAi. One group purposely used the transplastomic approach to preserve the intactness of the hpRNA^10^, while the other used short hairpins^12^ that were processed by some unknown mechanism within the chloroplast to give (presumably duplexed) siRNAs (Fig. 1). Experiments using tissue culture suggest that lepidopteran cells take up dsRNA but do not process it into siRNAs.^26^ This poses some intriguing questions: 1. Are the siRNAs derived from short hairpins able to escape from the endocytic vesicles and therefore interact and direct the lepidopteran RNAi machinery? 2. Do longer hpRNAs fortuitously have vesicle escape signals? 3. Do the chloroplast membranes, chloroplast membrane proteins, and/or constituents within the chloroplast affect the vesicular uptake and/or release of the hpRNA? If further research can answer these questions and provide the design rules to get si/ds/hpRNAs to the lepidopteran RNAi machinery, this may also provide the design for a wide range of insects. Unfortunately, it seems unlikely that cpTK‐RNAi will be useful for controlling aphids. Restricting the dsRNA to the chloroplast will prevent it being present in the plant's vasculature, and especially the phloem sap on which the aphids feed. Similarly, cpTK‐RNAi will probably not be very effective against root‐feeding pests unless pro‐plastids in the roots are sufficiently abundant and active.

Gene expression in chloroplasts can be polycistronic and and expression of up to 13 genes as a polycistronic cassette has been demonstrated to be possible.^84^ Once the design rules of cpTK‐RNAi are better established, this could be exploited to develop transplastomic plants expressing several insecticidal genes, targeting different sites within the insect pests. This could have the benefit of giving durable polygenic resistance. Indeed, with the advances of synthetic biology, it may be possible in the future to replace the entire genome of a chloroplast with a completely human‐designed chemically synthesized version. The concept was demonstrated with the replacement of the *Escherichia coli* genome with a synthetic version.^85^ Currently, synthetic plastomes would be prohibitively expensive, but with declining costs and rapidly advancing technologies it might not be so long before it becomes a realistic option. It would certainly be far easier and cheaper than replacing a plant's nuclear genome.

The current versions of cpTK‐RNAi have both advantages and disadvantages compared with nuclear‐encoded TK‐RNAi. They deliver higher levels of intact ds/hpRNA and stronger protection against leaf‐feeding insects than conventional TK‐RNAi. It remains to be elucidated which version is the best and how they each operate. Indeed, it may be that different nuances will be better suited to different pests. Finding an alternative to or a partner for Bt to protect crops against lepidopteran pests is a major goal and cpTK‐RNAi may go some way to providing this. However, the current versions only operate in green tissues and this restricts their application. A ubiquitous organelle, such as the mitochondrion, might provide the solution, if transformation protocols can be developed.
